# Depression among people living with HIV at a district hospital in KwaZulu-Natal, South Africa

**DOI:** 10.4102/safp.v66i1.5821

**Published:** 2024-07-22

**Authors:** Zothile L. Vezi, Selvandran Rangiah, Kantharuben Naidoo

**Affiliations:** 1 Department of Family Medicine, Faculty of Health Sciences, University of KwaZulu-Natal, Durban, South Africa

**Keywords:** HIV, AIDS, depression, prevalence, PLWHA, South Africa

## Abstract

**Background:**

Depression is a debilitating condition worldwide and a major contributor to the overall global burden of disease. The prevalence of depression is estimated to be higher in people living with HIV and AIDS (PLWHA) compared to the general population, with disease related complications increasing when mental health problems remain untreated. The aim of the study was to determine the prevalence of depression among PLWHA who attend a district hospital ART clinic in KwaZulu-Natal (KZN), South Africa (SA).

**Methods:**

Using strict systematic sampling, a descriptive cross-sectional study was employed with 121 adult outpatients attending an antiretroviral clinic at a district hospital in KZN, SA. Their biographical and clinical characteristics were obtained through a questionnaire and medical records, while depression was evaluated using the PHQ-9 scale. The data were analysed using descriptive and inferential statistics.

**Results:**

A total of 121 participants were recruited for this study. The prevalence of depression was 19.8% and significantly associated with a poor support system (adjusted odds ratio [aOR] = 3.60, *p* = 0.010). Female patients were more likely to have depressive symptoms than males (aOR = 0.73; confidence interval: 0.28–1.90) although this was not statistically significant. Age, marital status and viral load were not contributors to depression.

**Conclusion:**

Routine screening for depression among PLWHA at primary health care (PHC) level may improve detection rates, earlier treatment and overall health outcomes.

**Contribution:**

The results emphasise the need for patient-centeredness and holistic care that involves addressing mental health for PLWHA, given that HIV is a lifelong condition.

## Introduction

As of 2021, sub-Saharan Africa (SSA) remains the epicentre for human immunodeficiency virus (HIV), with 20.6 million people living with HIV and/or AIDS (PLWHA), and comprises 54% of the infection worldwide.^1^ In South Africa, 8.2 million people are estimated to be living with HIV, with KwaZulu-Natal (KZN) province having the highest incidence, estimated at 27% in 2017.^2^ The effectiveness of antiretroviral therapy (ART), notably triple therapy, and the growing availability of treatment centres have resulted in an increase in longevity and quality of life (QOL) in PLWHA.^2^ Correspondingly, a rapid rise in non-communicable diseases (including mental illness) has been observed globally, possibly influenced by changes in demographic and environmental factors that result from behavioural and nutritional changes.^3^

Mental health is recognised as an essential component of the state of well-being,^4^ the term Common Mental Disorder (CMD) being used to describe disorders that are highly prevalent in the general population, usually occurring at rates > 10%,^5,6,7^ and typically include depression, anxiety and substance use disorders.^5^ The prevalence of mental disorders in PLWHA has doubled globally,^5^ while in South Africa (SA), 26% – 38% of PLWHA have a CMD, compared to its estimated prevalence in the general population of 12.6%.^5,6^

Depression is a debilitating condition and a major contributor to the overall global burden of disease.^7,8^ The Diagnostic and Statistical Manual of Mental Disorders, fifth edition (DSM-5), defines depression as a mood disorder that comprises several physical, cognitive and emotional symptoms that impair an individual’s daily functioning.^9^ The prevalence of depression in the general population is estimated to be 10% – 15%, compared to 20% – 32% in PLWHA globally.^10^ In high-income countries, the estimated prevalence of depression among PLWHA is approximately 25%, which is three times higher than in HIV-uninfected individuals,^10^ while in SSA, it is estimated to be as high as 63%.^10^

In a cohort study in East Africa among 2300 participants, 18% – 25% met the Centre for Epidemiologic Studies Depression Scale (CESD) threshold for depression.^11^ In comparison, in Kalafong Provincial Hospital, South Africa, 53% of the 622 participants met the CESD threshold for depression.^12^ The proportion of PLWHA with depression may be even higher in low- and middle-income countries (LMICs), such as those in SSA, with its high burden of HIV infection, where mental healthcare services are sub-optimal.^10^

Human immunodeficiency virus and depression are both chronic illnesses that carry stigma and discrimination worldwide.^13^ While HIV can damage the subcortical areas of the brain that results in mood symptoms, depression can arise independently of HIV or can be a psychological consequence of being HIV infected.^13,14^ Studies have found that depression among PLWHA is likely to increase HIV transmission and decrease adherence to ART, leading to virological failure and rapid HIV progression.^12,13^ Not only does this perpetual cycle negatively impact on the patient’s QOL, it also increases the HIV and AIDS-related mortality rate.^7^ Effective treatments can reduce major depression in 80% to 90% in such patients.^10^

The challenges that relate to the poor recognition of depression in PLWHA include the social belief that it is acceptable for PLWHA to feel sad.^13^ This is compounded by the lack of integration in the management of HIV infection and mental illness at primary health care (PHC) level, where counselling focuses on the ability to adjust to a positive diagnosis and not on the long-term consequences, such as depression. Mental healthcare and HIV and AIDS, sexually transmitted infections and tuberculosis (HAST)-trained practitioners often struggle to manage the co-infection holistically because of the increasing workload, limited human resources, the lack of screening tools and a lack of inter-disciplinary training of both mental and HIV health.^13^

A recent study by Moosa et al.^15^ found that early detection of depression and its effective on-going management improve the adherence to ART.^15^ Their study aimed to determine changes and adherence to antiretroviral (ARV) drugs in HIV-positive patients with depression following treatment with an antidepressant and/or psychotherapy. Using the Hamilton depression rating scale, the depressed patients were randomly assigned to either taking anti-depressant or having interpersonal psychotherapy (IPT).^15^ At the end of the study, the mean ART adherence rate had increased to almost 100% for those who received pharmacotherapy (92.8% – 99.5%) and psychotherapy (91.1% – 99.6%).^15^

While effective treatments can reduce major depression in 80% to 90% of such patients,^10^ some ARVs have been associated with neuropsychiatric side effects, such as depression and anxiety, which may affect adherence.^16^ Efavirenz, which is a non-nucleoside reverse transcriptase inhibitor (NNRTI), and to a lesser extent dolutegravir, which is an integrase inhibitor, have been reported to produce neuropsychiatric symptoms, such as sleep disturbances, hallucinations and mood dysregulation. In South Africa, efavirenz has been reported to be one of the ingredients in a street drug called whoonga, because of its hallucinogenic property.^16^ In many developed countries, it is no longer considered a first-line choice for highly active antiretroviral therapy (HAART) because of its psychiatric side effects, which may limit patient compliance.^16^

The literature suggests that while the prevalence of depression is high among PLWHA, it is often unrecognised, with a paucity of local data among PLWHA. This study therefore aimed to establish the prevalence of depression and explore contributing factors in PLWHA attending a public sector district hospital in the eThekwini District, KwaZulu-Natal, South Africa.

## Methodology

### Design and setting

This cross-sectional descriptive study was conducted at the Masibambisane ARV clinic, which is located at the district level public sector Wentworth Hospital in Durban, between 01 August and 31 December 2022. The clinic offers specialist care in family medicine and is complemented by doctors, nurses, counsellors, a dietitian, social workers and a registered counselling therapist, providing services to 900 adults and 75 children monthly. It is a referral facility for patients with HIV and ART-related complications from local PHC clinics, the majority of patients being black, because of the catchment area it serves.

### Sample and procedure

Based on the number of patients seen monthly, the z test logistic regression was used to calculate a sample size of 108 patients. The statistical parameters used to calculate the sample size were an effect size = 3.5 (odds ratio) and type 1 (α) error = 0.05 (the probability of falsely rejecting the null hypothesis = 5%). Statistical power (1-β err prob) = 0.8 (Statistical power of 80%). When resources become available, the sample can be increased with a corresponding increase in power, but only to a maximum of 200, beyond which there will be no more improvement in the power.

Systematic random sampling was employed to identify potential participants, who needed to meet the inclusion criteria of ages 18 and older, enrolled in the Masibambisane ART programme for any length of time and taking ART, HIV-positive but ART naïve and able to provide written informed consent to participate in the study. Human immunodeficiency virus-positive patients who were minors, previously diagnosed with mood disorders, had recent bereavement, pregnant or could not consent to participate in the study were excluded.

The nursing and administrative staff were trained by the researcher to identify every third patient entering the ARV clinic, specifically those either visiting the clinic for ART initiation, follow-up with the physician or scheduled for blood sampling. The study was introduced to the potential participants by the trained nursing staff while they were awaiting service. Data were collected through face-to-face interviews after the required services had been provided to them, which resulted in their medical charts being tagged for identification and a copy of the biographical data collection sheet and Patient Health Questionnaire 9 (PHQ-9) questionnaire inserted. Informed written consent was obtained from each patient prior to the interviews, which were conducted in either English or IsiZulu, depending on the participant’s preference.

A structured data collection sheet was used on which to record their demographic data (age, gender, race, marital status, occupation, educational level and support system) and medical data (period on treatment, CD4 count, latest viral load and ART regimen). The PHQ-9 questionnaire, which is a self-administered tool, was completed with the aid of the researcher during the interviews to assess for depressive symptoms, with those who had a positive score of 10 and above, being further assessed for suicidal ideations. Participants with suicidal ideations would be admitted to the medical wards for psychosocial and pharmacological treatment. Depending on the severity of their depression, patients were referred to the treating doctors at Masibambisane for initiation of antidepressants. The drugs of choice were individualised to each participant because of drug–drug interactions between ARTs and antidepressants. Patients were given 2 weeks to 1 month follow-up depending on the severity of the depression. They were also referred to the social workers and a registered therapist for psychosocial treatment, coping mechanisms and psychotherapy.

### Measuring instruments: Patient Health Questionnaire 9

The PHQ 9, which is a validated multi-purpose instrument for screening, diagnosing, monitoring and measuring the severity of depression, incorporating (DSM-5) diagnostic criteria, was used to document depressive symptoms. The PHQ 9 uses a four-point Likert scale (0 = not at all, 1 = several days, 2 = more than half the days, 3 = nearly every day) to gauge responses to the questions asking about the respondents’ emotional health over the previous 2-week period. Scores on the PHQ 9 can range from 0 to 27;^17^ 0–4 indicating no depression, 5–9 mild, 10–14 moderate, 15–19 moderately severe and ≥ 20 severe depression. Scores of > 10 seldom occur in individuals with major depression, while scores of ≥ 15 usually signify the presence of major depression.^17^ For this study, 10 was used as the cut-off, as the tool has an overall sensitivity of 84% and a poor specificity of 72%, having been used in various studies in SA.^17,18,19^

The CESD has been validated in black undergraduate students in SA but has not been validated in the study population and was therefore not considered for this study.^20^

### Data management and analysis

The collected data were entered into an Excel spreadsheet and imported into Stata version 17 for analysis, the descriptive statistics being computed and reported as frequencies and proportions (%) for the categorical variables (e.g., age, race, viral load and CD4 count). The Pearson Chi-square (Chi^2^) or Fishers exact tests were used to determine factors associated with depression using bivariate analysis. Age, gender and viral load suppression were entered in the multivariate logistic regression model as confounders, while other factors were entered in the multivariable model if they had a *p* < 0.05 in the bivariate analysis. The outputs of the multivariate regression model are reported as adjusted odds ratio (aOR) and their corresponding 95% confidence interval (CI), with a *p* < 0.05 being considered statistically significant in the final model.

### Definitions used in the study

The cut-off for the PHQ 9 was 10, which meant that patients who scored 10 and below were regarded as not being depressed, but counselled extensively about depressive symptoms and encouraged to report if they manifested or worsening. Viral load was regarded as the number of viral particles in each quantity of blood drawn, with values of below < 50 copies/mL being considered virologically suppressed, between 50 and 1000 low viraemia, and above 1000 being virological failure. Drug regimens referred to first- or second-line ARV medication, with first line being a three-drug combination consisting of two nucleoside reverse transcriptase inhibitors (NRTIs) administered with a NNRTIs or an integrase inhibitor and second line being two NRTIs plus a ritonavir-boosted protease inhibitor. However, there are instances where protease inhibitors are used in the first line regimen, such as in rare cases where a patient cannot tolerate NNRTIs or NRTIs.

### Data storage and management

All digital data were encrypted and stored in a secure lock-up facility at the Department of Family Medicine at Wentworth Hospital, with key access only by the researcher. The hard copy sheets were scanned and stored with the digital data on a password-protected computer. Data will be stored for 5 years after the study and then destroyed.

### Ethical considerations

Ethical approval to conduct the study was obtained from the Biomedical Research Ethics Committee (reference number BREC/00004452/2022) of the University of KwaZulu-Natal (UKZN), Department of Health (DOH) and the Wentworth District Hospital. The study was conducted in accordance with SA DOH Research Ethics guidelines (2015) and the UKZN policy on research ethics. Patient participation was entirely voluntary; there was no reimbursement, incentive or coercing into participation, nor were they denied services if they refused, and only those who had signed a written informed consent were interviewed.

## Results

### Socio-demographic and clinical characteristics

A total of 121 participants were randomly selected and included in the final analysis; most (62/121) (52.9%) were female, black people (86%). Most of the participants were between the ages of 36–55 years (54.5%) and single (79/121) (65.3%). Though the majority had graduated high school (86/121) (71.1%), there was still a large number that was unemployed 47/121 (38.8%) (Table 1). Although the inclusion criteria for this study accepted individuals who are ART naïve, all of the participants sampled were already on treatment.

**TABLE 1:** Demographic and clinical characteristics of people living with HIV at Wentworth Hospital, KwaZulu-Natal, South Africa (August 2022 – December 2022).

Characteristic	Categories	*n*	%
Age (years)	18–35	29	24.0
	36–55	66	54.5
	≥ 56	26	21.5
Gender	Female	64	52.9
	Male	57	47.1
Race	Black people	104	86.0
	Other	17	14.0
Marital status	Divorced/separated	6	5.0
	Married	36	29.8
	Single	79	65.3
Education	No education or primary	16	13.2
	High school	86	71.1
	Tertiary	19	15.7
Employment	Pensioner	18	14.9
	Employed-skilled	37	30.6
	Students	5	4.1
	Unemployed	47	38.8
	Employed-unskilled	14	11.6
Support system	Good support	105	86.8
	No support	16	13.2
Treatment duration (years)	0–3	39	32.2
	4–6	25	20.7
	7 and above	57	47.1
CD4 count (cells/mL)	0–350	37	30.6
	350–500	28	23.1
	above 501	56	46.3
Viral load status	< 50 Copies	83	68.6
	50–1000 Copies	14	11.5
	> 1000 Copies	22	18.2
	Not yet due	2	1.7
ART regimen	NNRTI (EFV/NVP)	23	19.0
	Integrase Inhibitor	80	66.1
	2nd/3rd Line	18	14.9

NNRTI, Non-Nucleoside Reverse Transcriptase Inhibitor; ART, antiretroviral therapy; EFV, efavirenz; NVP, nevirapine.

Most of the participants (68.6%) were virally supressed with a viral load of < 50 copies. Two of the participants had no viral load result as they had not completed 6 months since initiation on ART (Table 1). Eighty (66.1%) participants were on the integrase inhibitor-based regimen, while 23 (19%) were on NNRTI efavirenz or nevirapine-based regimen.

### Prevalence

The prevalence of depression among PLWHA, as measured by the PHQ 9 questionnaire, was 19.8% (24/121) all of whom had a PHQ 9 score above 10, with moderate to severe depressive symptoms. While none had active suicidal ideations, eight met the criteria for further assessment and referral, as they had moderately severe to severe depressive symptoms scoring of 15–19 and 20–27 and were therefore referred to the social services department for further psychosocial assessment and/or psychiatric management.

A bivariate logistic regression analysis was used to determine the association between depression and the categorical variables, with no significant difference in depression status across different age groups (*p* = 0.468). The females had a higher percentage of depression (23.4%) than the males (15.8%), with the adjusted OR not being statistically significant (0.73, 95% CI: 0.28–1.90) (Table 2). Although the literature reports manifestations of neuropsychiatric symptoms secondary to certain ARV drugs, there was no statistical significance between the different ART regimens and major depression, and while the integrase inhibitors had a higher percentage (22.5%) compared to NNRTI (EFV/NVP) (17.4%), the *p*-value (*p* = 0.520) was not statistically significant.

**TABLE 2:** Prevalence of depression and associated factors (at bivariate analysis) among people living with HIV at Wentworth Hospital, KwaZulu-Natal, South Africa (August 2022 – December 2022).

Characteristic	Categories	Major depression				Total (*n*)	*P*
		No		Yes			
		*n*	%	*n*	%		
Age (years)	18–35 years	21	72.4	8	27.6	29	0.468
	36–55	55	83.3	11	16.7	66	-
	> 56	21	80.8	5	19.2	26	-
Gender	Female	49	76.6	15	23.4	64	0.292
	Male	48	84.2	9	15.8	57	-
Race	Black people	81	77.9	23	22.1	104	0.120
	Other	16	94.1	1	5.9	17	-
Marital status	Divorced/separated	3	50.0	3	50.0	6	0.120
	Married	31	86.1	5	13.9	36	-
	Single	63	79.7	16	20.3	79	-
Education	No education or primary	11	68.8	5	31.3	16	0.287
	High school	72	83.7	14	16.3	86	-
	Tertiary	14	73.7	5	26.3	19	-
Employment	Pensioner	14	77.8	4	22.2	18	0.805
	Employed-skilled	30	81.1	7	18.9	37	-
	Students	3	60.0	2	40.0	5	-
	Unemployed	39	83.0	8	17.0	47	-
	Employed-unskilled	11	78.6	3	21.4	14	-
Support system	Good support	88	83.8	17	16.2	105	**0.010**
	No support	9	56.3	7	43.8	16	-
Treatment duration (Years)	0–3	29	74.4	10	25.6	39	0.406
	4–6	22	88.0	3	12.0	25	-
	7 and above	46	80.7	11	19.3	57	-
CD4 count (Cells/mL)	0–350	26	70.3	11	29.7	37	0.190
	350–500	24	85.7	4	14.3	28	-
	above 501	47	83.9	9	16.1	56	-
Viral load status	< 50 copies	65	78.3	18	21.4	83	0.530
	50–1000 copies	13	92.9	1	7.1	14	-
	> 1000 Copies	17	77.3	5	22.7	22	-
ART regimen	NNRTI (EFV/NVP)	19	82.6	4	17.4	23	0.520
	Integrase Inhibitor	62	77.5	18	22.5	80	-
	2nd/3rd Line	16	88.9	2	11.1	18	-

Note: *p*-value, the bold values represent a significant finding.

NNRTI, non-nucleoside reverse transcriptase inhibitor; ART, antiretroviral therapy.

*, *p* < 0.05; **, *p* < 0.01; ***, *p* < 0.001.

A bivariate analysis of depression and its contributing factors is illustrated in Table 2.

### Association between depression and participant characteristics

Despite females having a higher prevalence of depression than males, there was no significant association between gender and depression, with age also not being significantly associated. Participants aged 36–55 years had a lower percentage of major depression (16.7%) compared to those aged 18–35 years (27.6%), but the adjusted OR was not statistically significant (0.73, 95% CI: 0.24–2.22). Participants aged > 56 years had a similar percentage of major depression (19.2%) compared to those aged 18–35 years, and the adjusted OR was also not statistically significant (0.97, 95% CI: 0.24–3.84).

*Support system* was significantly associated with major depression, with those having no support indicating a higher percentage of major depression (43.8%) than those with good support (16.2%), the adjusted OR being statistically significant (3.60, 95% CI: 1.11–11.71). Participants with no support were 3.6 times more likely to have depression than those with good support; however, because of the wide CI, this may not be a precise representation of the population. *Viral load* status was not significantly associated with depression, as participants with a viral load < 50 copies had a similar percentage of depression (21.4%) to those with a viral load > 50 copies (17.1%), the adjusted OR not being statistically significant (0.88, 95% CI: 0.29–2.66). The factors associated with depression among people living with HIV are illustrated in Table 3.

**TABLE 3:** Factors associated with depression among people living with HIV at Wentworth Hospital, KwaZulu-Natal, South Africa (August 2022 – December 2022).

Characteristic	Categories	*n*	%	Adjusted OR	95% CI
Sex	Female	15	23.4	1	
	Male	9	15.8	0.73	0.28–1.90
Age (years)	18–35	8	27.6	1	
	36–55	11	16.7	0.73	0.24–2.22
	> 56	5	19.2	0.97	0.24–3.84
Support system	Good support	17	16.2	1	
	No support	7	43.8	3.60*	**1.11–11.71**
Viral load status	Less than 50 copies	18	21.4	1	
	More than 50 copies	6	17.1	0.88	0.29–2.66

* , *p* < 0.05.

## Discussion

The main objective of this descriptive cross-sectional study was to determine the prevalence of depression among PLWHA at a district hospital in KZN as well as its contributing factors. The clinical characteristics of participants in this study included treatment duration, CD4 cell count, viral load and ART regimen, none of which were associated with depression. These findings are in contrast with Coppenhagen, whose participants with a low CD4 count, particularly less than 50, were found to have a higher rate of depression.^12^ The literature reports that certain ARV drugs, such as efavirenz, may cause mood dysregulation, and although only four (17.4%) participants out of the 24 were on an efavirenz-based regimen, there was no statistical evidence to suggest that the major depression was a result of this drug. As the new Dolutegravir (DGT)-containing ART guidelines in SA are rolled out, there are fewer patients remaining on EFV-based regimen. Though literature reports that DGT is also associated with neuropsychiatric side effects, a cohort study in Lesotho observed an improvement in symptoms such as feeling sad or depressed and anxiousness among participants who had transitioned from EFV to DTG.^21^ This study assessed the effects of transitioning from EFV to DGT-based ART on self-reported psychological and physical wellbeing. They used a PHQ-9 to screen for depression and found that at both baseline and follow-up, more than 93% of the participants had a PHQ-9 score indicating no or minimal depressive symptoms.^21^

The prevalence of depression in this study at Wentworth District Hospital that used the PHQ 9 questionnaire with cut-off of 10 was found to be 19.8%, indicating that one-fifth of the patients screened were depressed. The results were significantly lower than those of Coppenhagen, which reported 53% prevalence,^12^ with a study conducted at Kalafong Hospital, Gauteng province, in a specialised ART clinic with 622 participants. They used the CESD instrument to measure depression,^12^ the variations in the depression prevalence possibly being because of a larger sample size and the use of a different assessment instrument. The cultural and religious differences may also have contributed to the variations of depression between these two settings.^17^

This study showed some similarities in prevalence of depression to a national survey conducted in South Africa by Craig on 3402 participants across the nine provinces of South Africa using the PHQ 9 questionnaire.^22,23^ The prevalence of probable depression ranged from 14.7% to 38.8% and was estimated to be 14.9% in KZN.^22^ The findings of this is important for healthcare workers based in PHC institutions and indicate the need to integrate mental health and HIV health.^12,22^

The contention that depression affects more females than males^12,17,24^ is attributed to the multiple roles and responsibilities they carry,^17,25^ with factors such as unemployment, gender discrimination and violence significantly contributing towards women’s poor mental health.^25^ This is supported by Moshomo in a study in Botswana and Nyirenda in a South African study who assessed for depression prevalence among older people in rural areas.^24,25^

Although there were more female participants with depressive symptoms than males in this study, these findings are not statistically significant. This is supported by a study conducted at Kalafong Hospital in South Africa^12^ and further reiterates that depression may not be gender specific.^12,26^ Though South Africa has implemented different strategies to improve mental health in the country, there has not been a focus on routine screening at PHC facilities.

In this study, the age group 18–35 years had a higher percentage (27.6%) of major depression than other age groups, and despite it not being statistically significant, it suggests that young adults remain very vulnerable to the condition, these findings being similar to studies conducted elsewhere.^11,26^ However, these findings are in contrast with a study conducted in rural Northern KZN, where a higher prevalence of depression was found among older people infected and affected by HIV and AIDS.^25^ Although the criteria included adolescents aged between 18 and 19 years in this current study, none of the participants were of this age, possibly because of the time period in which the data collection process was conducted as it coincided with examinations in both schools and tertiary learning institutions.

The bivariate analysis revealed that marital status, employment status and education level were not significantly associated with depression, with other studies having reported that a higher level of education decreased the risk of depression.^24^ Worldwide, the association between marital status and increased risk of depression has been observed to be marginally higher in single individuals, but no statistical difference was found.^24^ In contrast, a cross-sectional study in South Africa found that both probable depression and anxiety were significantly increased in divorced, separated and widowed individuals,^22^ this being attributed to the psychological and financial stress brought about by the disruption of the marriage.

A poor social support system was statistically significant in this study, with the odds of having depression being significantly increased in patients who had no support system (aOR = 3.60, 95% CI: 1.11 – 11.71). These findings are consistent with a systemic review and meta-analysis by Weldesenbet in East Africa,^26,27^ where eight studies conducted in Ethiopia that explored the effects of poor social support on depression among PLWHA^26^ found that PLWHA who had no social support were more likely to develop depression.^26,27,28^

The lack of social support remains a risk factor for developing and/or having depression among PLWHA, possibly as they are more susceptible to stigma and discrimination associated with the disease.^27,28^ Their employability is impacted by their HIV status, because of the burden of disease and stigmatisation in the workplace, and recurrent ARV clinic visits being a significant ongoing financial commitment. Furthermore, this could be compounded by personalised stigma, negative self-imaging and public attitude,^28^ which result in help seeking avoidance, isolation, loneliness and increased vulnerability to depression.

The effects of depression among PLWHA can be detrimental to their health, with the integration of mental health, psychological and psychosocial services into HIV and AIDS care possibly resulting in better outcomes for those at greatest risk.^29^ This is confirmed by a study that examined 109 participants who were HIV-positive with depressive symptoms in a randomised control trial in Uganda.^29^ The participants were assigned to receive 8 weekly sessions of either Group Support therapy (GSP) or Group HIV Education (GHE), which were facilitated by trained mid-level mental health workers, the former offering skills about changing attitudes and practising positive social skills and coping strategies to reduce stigma. The author established that there was a reduction in depression among the GSP participants at 6 month review and concluded that increasing social support may mitigate stressors, such as stigma and maladaptive coping behaviour, to alleviate depression.^28,29^

### Study limitations

This study took place in a single clinical site with a very small sample size, thereby limiting generalisability to all PLWHA in KZN. Although the study gives some indication of the current clinical characteristics of PLWHA, it is urban and hospital based, which means that the findings may not be generalisable to other settings, such as private sectors and rural areas with no comprehensive HIV care.^17,24^ While the literature suggests that there is a high prevalence of depression among the Lesbian, Gay, Bisexual and Transgender (LGBT) community living with HIV and AIDS,^30^ this study did not classify these genders. People living with HIV and AIDS often have multiple comorbidities such as diabetes mellitus, hypertension and opportunistic infections, which have been suggested to be predispositions to depression. A study in a specialist clinic in KZN found a depression prevalence of 36% among diabetic patients living with and without HIV.^17^ The current study failed to establish the association of concurrent comorbidities and depression. Another limitation was the use of the PHQ 9 questionnaire; although completion was facilitated by the primary investigator, it is prone to bias, which may lead to high false negative reporting.^19^ Despite this being one of the few studies exploring depression among PLWHA in a district level facility in KZN, further studies on this topic would benefit the body of knowledge for the entire health system.

## Conclusion

The prevalence of depression among PLWHA cannot be ignored in PHC settings, the condition being fairly common and under-recognised in PLWHA by both patients and healthcare practitioners. The study highlights poor or no support system as a significant associated factor and re-emphasises the need for a comprehensive contextual assessment and management of all patients, which is a fundamental family medicine principle. Further research is required to integrate mental health and HIV prevention and promotion in PHC, with additional psychosocial support also being a necessity for communities to improve their overall mental and physical outcome, as well as their QOL. Training of healthcare workers on mental health and providing resources such as screening tools at PHC level remains essential.
